# Enhanced production of recombinant serratiopeptidase in *Escherichia coli* and its characterization as a potential biosimilar to native biotherapeutic counterpart

**DOI:** 10.1186/s12934-019-1267-x

**Published:** 2019-12-17

**Authors:** Vishal Srivastava, Shivam Mishra, Tapan K. Chaudhuri

**Affiliations:** Kusuma School of Biological Sciences, Indian Institute of Technology Delhi, Hauz Khas, New Delhi, 110016 India

**Keywords:** *Serratia marcescens*, Serratiopeptidase, *Escherichia coli*, Metalloprotease, Inclusion bodies, Heterologous protein expression, Recombinant mature version serratiopeptidase (rMSrp)

## Abstract

**Background:**

*Serratia marcescens,* a Gram-negative nosocomial pathogen secretes a 50 kDa multi-domain zinc metalloprotease called serratiopeptidase. Broad substrate specificity of serratiopeptidase makes it suitable for detergent and food processing industries The protein shows potent anti-inflammatory, anti-edemic, analgesic, antibiofilm activity and sold as an individual or fixed-dose enteric-coated tablets combined with other drugs. Although controversial, serratiopeptidase as drug is used in the treatment of chronic sinusitis, carpal tunnel syndrome, sprains, torn ligaments, and postoperative inflammation. Since the native producer of serratiopeptidase is a pathogenic microorganism, the current production methods need to be replaced by alternative approaches. Heterologous expression of serratiopeptidase in *E. coli* was tried before but not found suitable due to the limited yield, and other expression related issues due to its inherent proteolytic activity such as cytotoxicity, cell death, no expression, minimal expression, or inactive protein accumulation.

**Results:**

Recombinant expression of mature form serratiopeptidase in *E. coli* seems toxic and resulted in the failure of transformation and other expression related issues. Although *E. coli C43(DE3)* cells, express protein correctly, the yield was compromised severely. Optimization of protein expression process parameters such as nutrient composition, induction point, inducer concentration, post-induction duration, etc., caused significant enhancement in serratiopeptidase production (57.9 ± 0.73% of total cellular protein). Expressed protein formed insoluble, enzymatically inactive inclusion bodies, and gave 40–45 mg/l homogenous (> 98% purity) biologically active and conformationally similar serratiopeptidase to the commercial counterpart upon refolding and purification.

**Conclusion:**

Expression of mature serratiopeptidase in *E. coli C43(DE3)* cells eliminated the protein expression associated with toxicity issues. Further optimization of process parameters significantly enhanced the overexpression of protein resulting in the higher yield of pure and functionally active recombinant serratiopeptidase. The biological activity and conformational features of recombinant serratiopeptidase were very similar to the commercially available counterpart suggesting it-a potential biosimilar of therapeutic and industrial relevance.
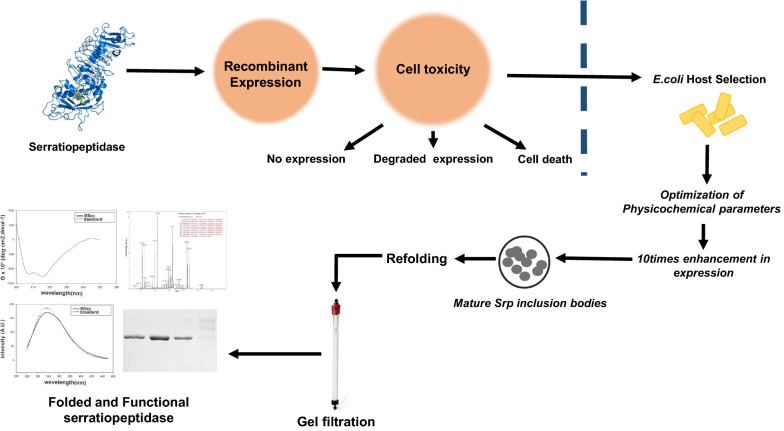

## Background

Proteases are one of the most abundant protein family represented by those protein molecules which hydrolyze substrate protein molecules by disruption of the peptide bonds in between constituent amino acids [1]. Extracellular microbial proteases are pivotal for the growth and survival of protease producing microrganisms [2] as they do protein catabolism in the surrounding environment producing smaller peptides or amino acids to fulfill the nutritional requirements of source organism [3]. Proteases act as virulence factors and are critical for initiating as well as establishing microbial infections. Apart from carrying out crucial biological functions, proteases are equally relevant in a variety of commercial and industrial applications such as—an additive in detergents, in food processing (meat tenderization, milk coagulation), brewing, leather tanning and paper industry [4–6]. The hydrolytic activity of proteases is equally relevant for therapeutic purposes. They are used as an oral digestive aid, local clearing agents for solubilizing protein deposits, minimizing inflammation, or as a thrombolytic agent in thromboembolic disorders [7]. Food and Drug Administration (FDA) approved at least 12 proteases as a drug for treating disorders like—hemophilia, stroke, AMI (acute myocardial infarction), unwanted inflammatory response, and digestive disorders [8–11]. Such a wide variety of industrial and therapeutic applications account for around 60% of worldwide enzyme sales attributed alone to proteolytic enzymes [12].

*Serratia marcescens*, a Gram-negative opportunistic pathogen secretes at least four different types of proteases. The majority of the proteolytic activity exhibited in the extracellular secretion was attributed to a 50 kDa zinc metalloprotease known as serralysin, serrapeptase, or serratiopeptidase [13–15]. Serratiopeptidase shows multidomain architecture containing a zinc atom in its catalytic site located in the N-terminal domain. The C-terminal domain of the protein consists of repeat-in toxin (RTX) glycine-aspartate rich motifs responsible for the binding of seven calcium atoms to the protein [16]. Broad specificity of serratiopeptidase is essential for the protein as a virulence factor to exhibit cytotoxicity and immunomodulation in a variety of hosts [17–21]. The broad specificity of serratiopeptidase is equally important for industrial applications such as—a detergent additive, in food processing, brewing, leather, and paper industry [22, 23]. Serratiopeptidase shows the potent anti-inflammatory and analgesic activity of therapeutic relevance and sold in the market either as a single component or as fixed-dose combination (FDC) enteric-coated tablets. The drug is prescribed for treating disorders like—chronic sinusitis, post-traumatic swelling, fibrocystic breast disease, bronchitis, healing after molar extraction, and post-surgical inflammation in several Asian and European countries [24]. The anti-inflammatory action of serratiopeptidase is attributed to its ability to break down insoluble protein exudates, facilitating drainage, and hydrolyzing inflammatory protein molecules [25]. The analgesic action of serratiopeptidase possibly functions through inhibiting the release of pain-inducing amines [26]. Serratiopeptidase is a potent anti-biofilm molecule and also disrupts amyloid fibrils in vitro as well as in vivo [27, 28].

The industrial and pharmaceutical demand of serratiopeptidase is fulfilled through growing wild and mutant strains of *S. marcescens* in nutrient-rich growth medium and further extracting the protein out from the extracellular broth. The present approach of production is source organism dependent and provides a narrow scope of optimization, hence also limiting the yield [22]. The pathogenic nature of the source organism and its association with a variety of infection ensures the need for an alternative approach for serratiopeptidase production. *Serratia marcescens* associated infections include but are not limited to ventilator-associated pneumonia, endocarditis, bacteremia, post-surgical infections, microbial keratitis, urinary tract infection, meningitis and necrotizing fasciitis [13, 17, 18, 29, 30]. Multi-drug resistant strains of *S. marcescens* are associated with clinical outbreaks in intensive and neonatal care unit are in the high priority list of World health organization (WHO) for developing novel antimicrobial therapies [31, 32]. Bulk release of bacterial biomass is a common thing during large-scale production of serratiopeptidase and potentially hazardous for associated people with industrial operations. Recombinant expression of serratiopeptidase in *E. coli* based system seems to be a viable solution that will not only limit the use of native pathogenic source strain but also provide an opportunity of various expression parameters. Optimization of expression parameters would result in enhancement in yield and even might prove cost-effective.

*Escherichia coli* cells are well studied, and a variety of engineered expression strains of *E. coli* are available. It also has a considerably fast growth rate and fermentation batch turnaround number equal to 300 per year, which is far higher than any of the host systems available [33]. *E. coli* are nutritionally versatile and in combination with the above-mentioned properties fit most suitable system for heterologous protein expression. *E. coli* based expression systems are used for recombinant production of around 30% FDA approved therapeutically relevant protein molecules; viz—human insulin, plasminogen activator, growth hormone [34]. Even after having such versatility, and *Serratia* protease genes cloned nearly 30 years ago [35] industries prefer the wild source organisms over *E. coli* based expression. The answer lies in the fact that *E. coli* based heterologous expression of proteases causes critical cellular stress due to the associated catalytic activity of proteases and failure of the expression system [36]. Sign of failure of expression system is often visualized in the form of cell lysis, growth inhibition, instability of the expression plasmids, lack of protein expression, degraded protein expression, or deposition of the proteins into non-functional misfolded aggregates; i.e., inclusion bodies [37].

The present work demonstrates the successful execution of an *E. coli* based alternative method for serratiopeptidase production in its propeptide devoid mature form. The expression was carried out in *E. coli BL21 C43(DE3)* cells designed explicitly for membrane and toxic protein expression. A significant enhancement in protein expression was achieved through the optimization of expression parameters such as growth medium, induction point, inducer concentration, temperature, and duration of induction. The protein expresses in the form of insoluble non-functional inclusion bodies, which were further refolded and purified into its functionally active folded form. The protein shows the activity, nature, and conformational features very similar to the commercially available native version of the protein. The molecule could be a recombinant biosimilar of serratiopeptidase for therapeutic purposes and industry-relevant applications.

## Results

### Recombinant cloning and development of mature serratiopeptidase expression construct (pMSrp)

Formation of transparent halo around the point inoculated culture (~ 1 × 10^6^ CFU) of the bacteria was attributed to the presence of extracellular proteases (shown in Additional file 1: Figure S1a panel-ii). There was a prominent protein band visible around 50 kDa molecular weight in SDS-PAGE gel, lane loaded with extracellular supernatant from 48 h grown bacterial culture (shown in Additional file 1: Figure S1b). Peptide mass fingerprinting of the corresponding protein band after trypsin digestion showed 51% sequence coverage with serralysin protease, also known as serratiopeptidase (shown in Additional file 1: Figure S2).

**Fig. 1:**
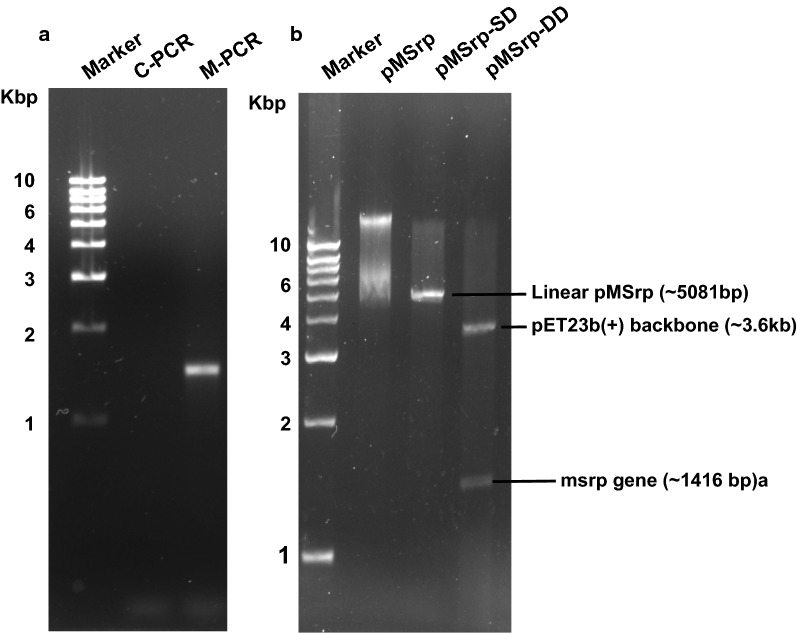
Recombinant cloning and development of mature serratiopeptidase specific expression construct. **a** Representative Agarose gel (1.2%) showing amplification of ~ 1500 bp gene fragment, particular to the size of mature serratiopeptidase gene (M-PCR). The gene cloned in the pET23b(+) vector having Amp^r^ for selection. When digested with single restriction enzyme; i.e. *Nde*I and two different enzymes; i.e., *Nde*I and *Xho*I. **b** Representative agarose gel (1.2%) shows a linear fragment (pMSrp-SD) of ~ 5000 bp and two fragments equal to the size of plasmid backbone ~ 3600 bp and insert gene (MSrp) ~ 1500 bp in Lane (pMSrp-DD) respectively confirming the successful insertion/ligation of gene and construction of recombinant plasmid

PCR amplified gene-specific to mature serratiopeptidase was 1416 bp in size and ligated downstream to T7 promoter in pET23b(+) expression vector in between *Nde*I and *Xho*I restriction sites. The developed recombinant plasmid was termed pMSrp. Single digestion of recombinant plasmid pMSrp generated linearized vector pMSrp-SD and confirmed the size of the recombinant construct equivalent to 5081 bp (Fig. 1b MSrp-SD) while the double digestion with *Nde*I and *Xho*I resulted in two linear fragments of around 3600 bp and 1416 bp equivalent to the vector backbone and the serratiopeptidase (srp) gene insert respectively (Fig. 1b Msrp-DD). Sequencing results along with restriction digestion results confirmed the successful cloning of the gene in pET23b(+) vector and development of recombinant expression vector pMSrp.

### pMSrp expression seems toxic for *E. coli* cells, and only *E. coli C43(DE3)* cells expressed the protein correctly

**Fig. 2:**
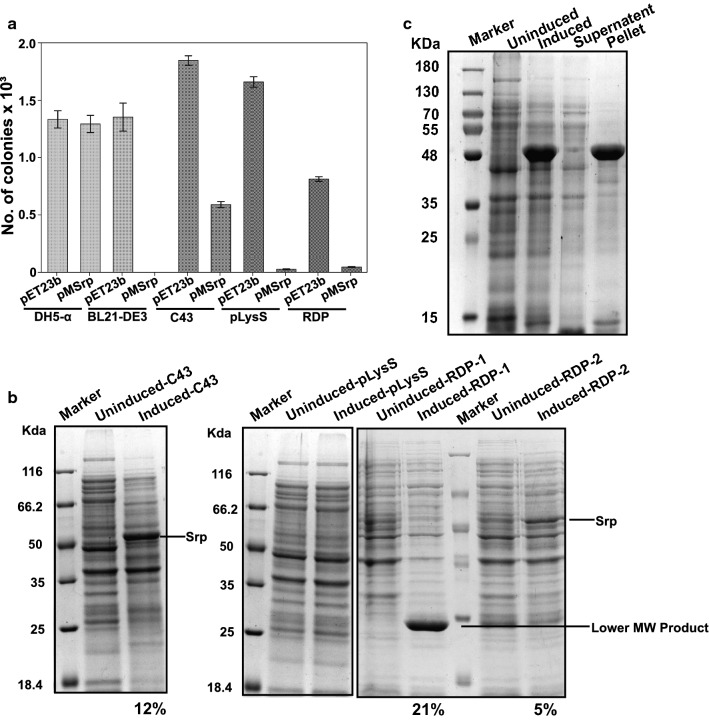
Selection of *E. coli* expression host system and expression of mature serratiopeptidase. **a** Bar graph showing the number of pMSrp plasmid transformants in different *E. coli* expression host systems. DH5-α, BL21-DE3, C43, pLysS and RDP represents the *E. coli* cloning and expression strains *E. coli DH5*-*α, E. coli BL21 (DE3), E. coli C43(DE3), E. coli BL21 (DE3)*-*pLysS,* and *E. coli Rosetta (DE3)*-*pLysS* respectively. **b** Representative SDS-PAGE gel lanes are showing expression of mature serratiopeptidase gene product in different *E. coli* expression systems. While *E. coli C43(DE3)* cells express protein at the correct size, in other systems, there is either no visible expression (*Induced*-*pLysS*) or degraded expression (*Induced*-*RDP*-*1*) unless induced at optical density > 1.0 at 600 nm (induced-RDP-2). The percentage contribution of the mature serratiopeptidase in total expressed protein either at correct molecular weight or in the degraded form is mentioned below the gel lanes. **c** Representative 12% SDS-PAGE gel showing total cell lysate of uninduced and induced cell fractions of *E. coli C43(DE3)* cells showing overexpression of protein equivalent to 50 KDa. Fractionated samples of cell lysate loaded on SDS-PAGE shows the mature serratiopeptidase expresses in the form of insoluble inclusion bodies and completely goes into the insoluble fraction; i.e., pellet

The number of transformed cells in the presence and absence of serratiopeptidase gene could explain the toxicity of the gene. The number of transformants in *E. coli DH5*-*α* after transformation with pET23b(+) (no gene) and pMSrp (mature gene) were almost equal but were significantly different in other DE3 variants of *E. coli*, suggesting that the presence of gene had some deleterious effect on cells. Transformation of the plasmid pMsrp was not possible in *E. coli BL21(DE3)*cells despite repeated attempts, as shown in Fig. 2a. While in other DE3 variants, viz—*C43(DE3), pLysS*, and *Rosetta(DE3)*-*pLysS* (RDP) number of successful transformants were significantly lesser than the vector alone (Fig. 2a).

When the protein expression was analyzed in different DE3 expression strains, the difference in protein expression was evident. Even after successful transformation, no visible protein expression was seen in *E. coli BL21 (DE3)*-*pLysS* cells (*pLysS*) as visualized on SDS PAGE (Fig. 2b induced *pLysS* lane). *E. coli Rosetta (DE3)*-*pLysS* cells (RDP) overexpressed a protein lesser than the actual size of mature serratiopeptidase, i.e., 50 kDa (Fig. 2b induced RDP-1 lane). Delayed induction of RDP cells; i.e., after OD_600_ > 1.0 resulted in the expression of the correct size protein representing ~ 5% of total protein expression (Fig. 2b induced RDP-2 lane) in comparison to degraded overexpressed protein which was around 21% of total protein expression in the cell lysate. The maximal transformation was found in *E. coli C43(DE3)* cells (Fig. 2a pMSrp in C43). The cells were expressing the protein at correct size and were around 12% of the total protein (Fig. 2b induced C43 lane). Fractionation assay results shown in Fig. 2c confirmed the overexpressed protein corresponding to the mature serratiopeptidase expressed in the form of insoluble inclusion bodies and goes entirely in the pellet fraction,

### Optimization of expression parameters resulted in five-time more expression of mature serratiopeptidase in *E. coli* cells

**Fig. 3:**
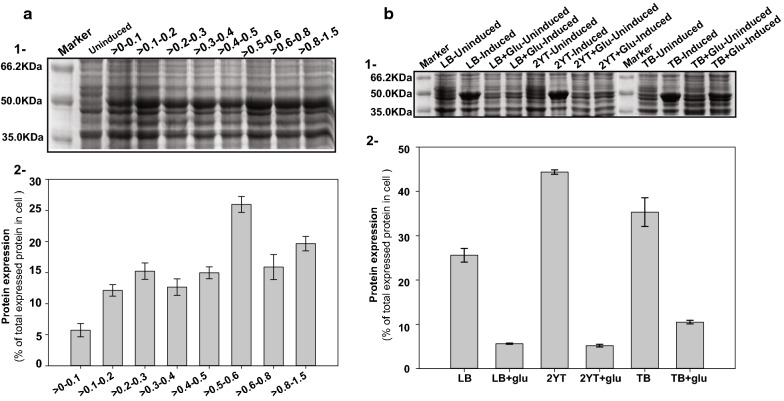
Optimization of serratiopeptidase expression in *E. coli C43(DE3)* cells. **a** Effect of induction on overexpression of mature serratiopeptidase in *E. coli C43(DE3)* cells at different time points of growth was observed by supplementing culture with 1 mM IPTG. Section 1 shows the SDS-PAGE gel loaded with induced samples at different time points. Section 2 shows the bar graph plot representing the relative expression percentage of mature serratiopeptidase as the average of three independent densitometric analysis. Section 1 of part **b** shows the overexpression profile of the recombinant version of mature serratiopeptidase in different growth media on 12% SDS-PAGE gel. Obtained percent values of serratiopeptidase expression in terms of total intracellular protein expression was averaged out for three independent densitometric analysis and used for plotting the bar graph shown in Section-2 of part-**b**

Without optimization, mature serratiopeptidase overexpression constituted about 12% of total protein expression in the form of insoluble inclusion bodies. Optimization of various physicochemical parameters was carried out with a hope of enhanced expression of recombinant protein. The point of induction was taken as first parameter for optimization, and this optimization alone enhanced the total cellular expression of mature serratiopeptidase by 1.46 times, the expression obtained at optical density ~ 0.6–0.8. The total protein expression was 25.9% at the optical density of 0.5–0.6 in comparison to 17.7% at 0.6–0.8 optical density measured at 600 nm (Fig. 3a, Sections 1 and 2).

**Fig. 4:**
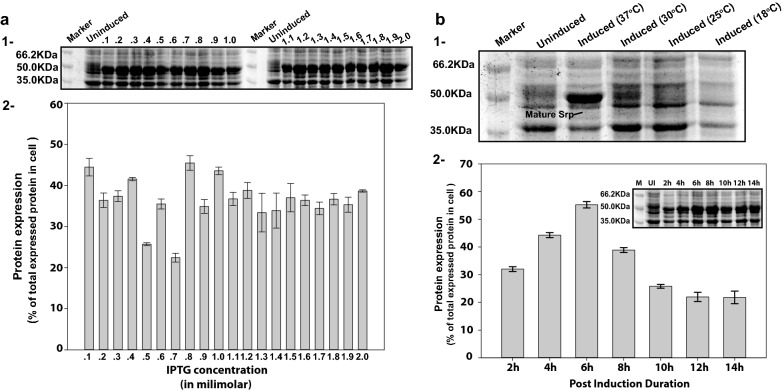
Effect of Inducer (IPTG) concentration, temperature, and post-induction duration on serratiopeptidase expression in *E. coli C43(DE3)* cells. **a** Representative 12% SDS-PAGE (section-1) gel showing the effect of inducer concentration on recombinant serratiopeptidase expression at different concentrations of inducer ranging from 0.1 to 2.0 mM. Section 2 of the image shows the plotted bar graph of average protein expression at different inducer concentrations obtained from the densitometric analysis of three independent SDS-PAGE gels. **b** Section 1 represents a 12% SDS-PAGE gel showing the effect of temperature on mature version serratiopeptidase under uninduced condition (uninduced lane) while other lanes represent induced cell lysates at different temperatures viz—37 °C, 30 °C, 25 °C, and 18 °C. Section 2 is the plotted bar graph showing the effect of post-induction duration on the expression of mature recombinant serratiopeptidase. The expression was measured from three independent sets of experiments and represented in the form of the average value of them, showing the percentage expression of mature serratiopeptidase in terms of total intracellular protein expression visualized on the SDS-PAGE gel

Effect of nutrient medium composition on mature serratiopeptidase overexpression was measured by analyzing the expression in three complex nutrient media compositions viz—Luria broth, 2YT broth, and Terrific broth. 2YT broth showed the maximum expression, comprising 44% of the total expression. The addition of glucose at 1% (w/v) negatively affected the level of protein expression, irrespective of the growth media composition (Fig. 3b, Sections 1 and 2). The next parameter chosen for an optimization was inducer concentration. RNA polymerase found in T7-promoter based vectors is lactose inducible. IPTG, a synthetic structural analog of lactose is preferred over lactose since it cannot be metabolized, so the concentration of inducer remains constant throughout the induction. The overexpression of mature serratiopeptidase varied from 25 to 44% at different concentrations of inducer ranging between 0.1 and 2 mM (Fig. 4a, Section 1). The maximum recombinant protein expression was observed at 0.8 mM, which was 45.4 ± 1.76% of total protein expressed (Fig. 4a, Section 2). Change in temperature does not have any significant effect on protein expression or solubility. Less expression of mature serratiopeptidase was visible at 30 °C in comparison to 37 °C. Induction at 25 °C or 18 °C shown no visible expression of recombinant protein when observed on SDS-PAGE, as evident in Fig. 4b, Section 1. The optimal temperature for serratiopeptidase expression was 37 °C, where expressed mature serratiopeptidase constituted about 45% of total protein expression. Post-induction duration for maximal expression was optimized between 2 and 14 h. The maximal expression of recombinant mature serratiopeptidase was found after 6 h of induction contributing 57.9 ± 0.73% of total intracellular protein expression (Fig. 4b, Section 2).

### The presence of plasmid pMSrp slowed down the growth rate of *E. coli* cells

**Fig. 5:**
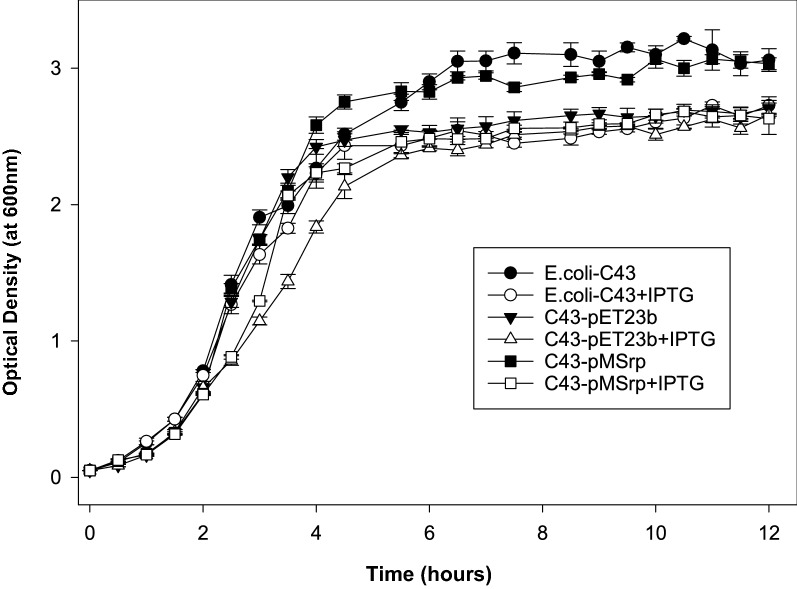
Effect of inducer and different vector constructs on growth kinetics of *E. coli C43(DE3) cells*

Bacterial cells transformed with plasmid-irrespective of its type or gene insert show differences in growth rate in comparison to non-transformed cells. These differences are resultant of altered internal energetics of the bacterial cells and affect the production rate of recombinant proteins. The growth kinetics of *E. coli C43(DE3)* cells was observed in the presence and absence of mature serratiopeptidase specific gene under uninduced as well as induced conditions. The *E. coli C43(DE3)* cells without any presence of plasmid as well as inducer were taken as a negative control. The obtained growth curve of *E. coli C43(DE3)* cells for 12-h duration, as shown in Fig. 5, was used for measuring the specific growth rate of the bacterium in different conditions. All the transformed cells showed a decrease in growth rate as compared to the wild-type *E. coli C43(DE3)* cells. It was also evident that the presence of IPTG as inducer significantly decreased the growth rate. The calculated specific growth rate of *E. coli C43*-*de3* cells in each condition are summarized in Table 1.

**Table 1:** Specific growth rate constants (µ) for the growth of *E. coli C43(DE3)* cells under different conditions at 37 °C

Conditions	Specific growth rate (µ) (h^−1^)
*E. coli C43(DE3)* cells	0.5604
*E. coli C43(DE3) *+ IPTG	0.6044
*E. coli C43(DE3)*-pET23b	0.5464
*E. coli C43(DE3)*-pET23b + IPTG	0.6091
*E. coli C43(DE3)*-pMSrp	0.5965
*E. coli C43(DE3)*-pMSrp + IPTG	0.7091

### Refolding and purification of isolated inclusion bodies provided homogenous enzymatically active serratiopeptidase

**Fig. 6:**
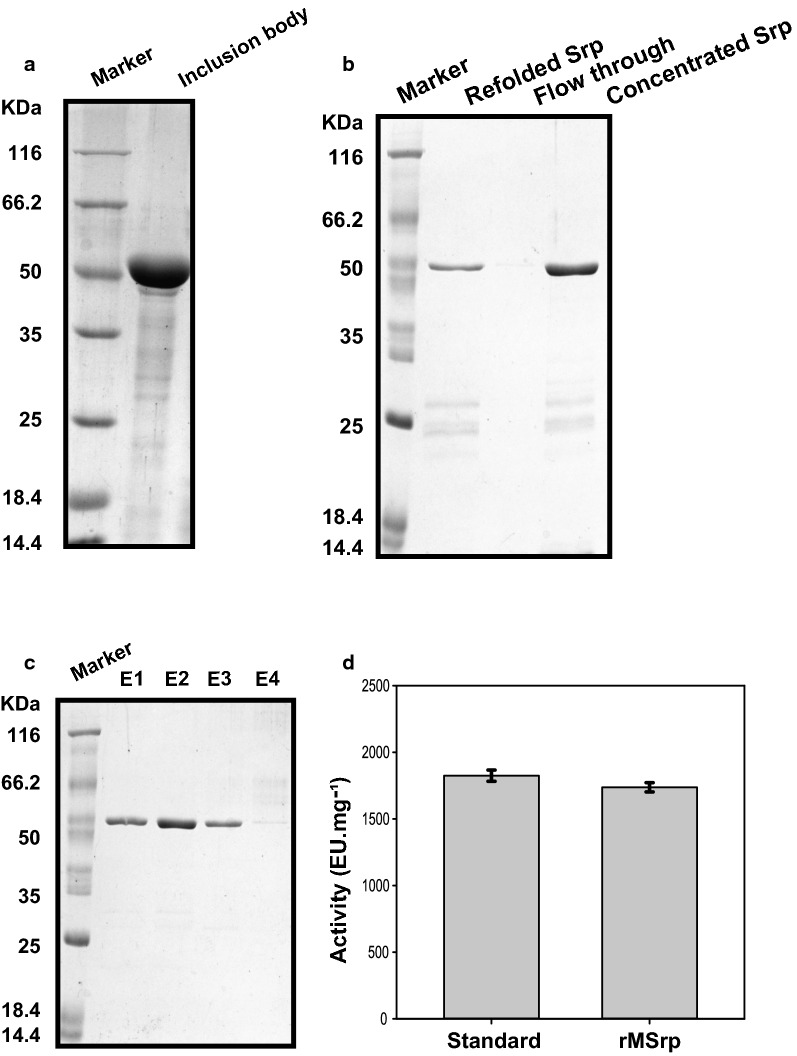
Purification of recombinant mature serratiopeptidase and proteolytic activity assay. Representative SDS-PAGE gels showing **a** isolated inclusion body (IB) of recombinant version mature serratiopeptidase from 6 h grown induced culture of *E. coli C43(DE3*) cells harbouring the expression plasmid pMSrp, **b** refolded serratiopeptidase by rapid dilution in ice-cold refolding buffer (refolded srp lane) and protein profile after concentration (concentrated srp). The lane Flow through was loaded with filtrate collected during concentration using 30 kDa molecular weight cut-off during concentration. **c** Representative SDS-PAGE gel showing collected elution fractions (E1–E4) of purified refolded mature version recombinant serratiopeptidase by size exclusion chromatography. **d** Protease activity of purified mature recombinant serratiopeptidase (rMSrp) its commercially available wild counterpart (standard) was measured using azocasein as substrate. The obtained specific activity of each one is plotted in the form of bar graph with error bars representing the standard error calculated from three independent experiments

125–135 mg serratiopeptidase inclusion bodies were obtained from a liter of grown culture, which was about 55% pure. Only the washing of inclusion bodies provided a 10% enhancement in the purity level, giving 100–120 mg of 60–65% pure inclusion bodies (Fig. 6a). The refolding efficiency of the protein was around 50%, and 55–60 mg refolded and enzymatically active recombinant mature serratiopeptidase was obtained after the rapid dilution (1:100) and concentration. The protein prepared through refolding has purity around 85–90% measured through SDS-PAGE (Fig. 6b). The trace amount of denaturants and protein contaminants were removed through size-exclusion chromatography, providing a yield of 45–50 mg > 98% pure functionally active recombinant mature serratiopeptidase (Fig. 6c). The yield measurement of enzymatically active refolded recombinant mature serratiopeptidase from 1 l of bacterial culture as measured by Bradford assay, densitometric analysis, and activity assay are given below in Table 2.

**Table 2:** Protein yield comparison at different steps of purification of the recombinant version of mature serratiopeptidase

Step	Yield (mg)	Purity (%)	Activity (EU/mg)
Isolation	130 ± 5	55	Not measured
Washing	110 ± 10	60–65	Not measured
Refolding by dialysis	57.5 ± 2.5	85–90	1650 ± 5 EU/mg
Gel filtration	47.5 ± 2.5	> 98	1750 ± 5 EU/mg

Azocasein based proteolytic assay suggests the specific activity of purified refolded recombinant mature serratiopeptidase was 1750 ± 5 EU/mg in comparison to commercial standard showing ~ 1820 ± 5 EU/mg (Fig. 6d).

### Recombinant version mature serratiopeptidase could be a potential biosimilar to the native counterpart of the protein

**Fig. 7:**
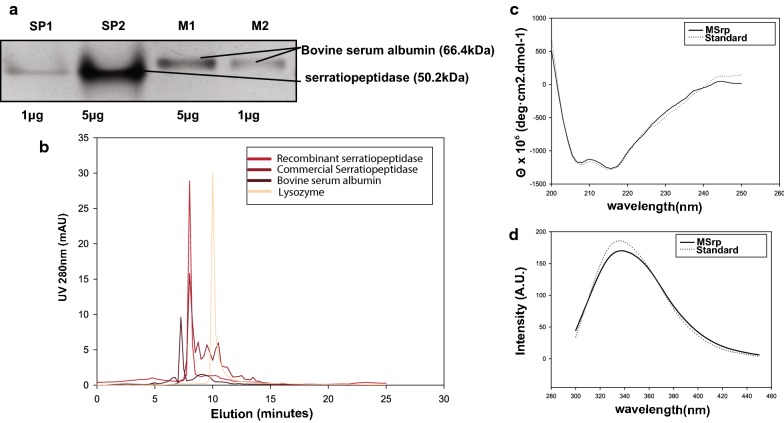
Biophysical characterization of recombinant mature version serratiopeptidase. **a** Native PAGE loaded with 1 μg and 5 μg of recombinant mature version serratiopeptidase (SP1, SP2) and bovine serum albumin (BSA) as a marker (M1, M2) suggests the purified protein is monomeric. **b** Analytical HPLC of the recombinant mature form, when compared to the commercial version serratiopeptidase and other control proteins viz—lysozyme (14.4 KDa) and bovine serum albumin (66.4 KDa) shows the protein elutes at 7.5 min coinciding with the significant peak of commercial version serratiopeptidase. The elution profile of the recombinant version is identical to the commercial counterpart, except there are very less intensity minor peaks in comparison to the commercial version. It suggests the protein does not have or very fewer contaminants or degraded products, if any, in comparison to the commercial counterpart. Comparative **c** circular dichroism spectra (200–250 nm) and **d** intrinsic protein fluorescence spectra(300–450 nm) of recombinant version mature serratiopeptidase (solid line) and its commercial counterpart (dotted line) showing both versions have an almost identical conformational signature, and recombinant version may prove a better biosimilar for application purposes

Native-PAGE, along with analytical HPLC results, concluded purified recombinant mature serratiopeptidase was homogenous preparation of functionally active monomeric molecules (Fig. 7a, b). The elution peak of recombinant preparation coincided with the commercial version standard eluting at 7.5 ml.

In-gel trypsin digestion and peptide mass fingerprinting analysis of generated peptide fragments matched with *S. marcescens* serratiopeptidase protein having a sequence coverage of 53% (shown in Additional file 1: Figure S3). Circular dichroism (CD) spectra, as well as intrinsic fluorescence spectra of recombinantly prepared mature serratiopeptidase, were very similar to the commercially available serratiopeptidase (Fig. 7c). Similarly, the intrinsic fluorescence emission maxima were 339 and 338 nm for mature recombinant version and the commercial standard respectively and were identical, as shown in (Fig. 7d).

## Discussion

Considering the therapeutical and industrial importance of serratiopeptidase [22, 24], in the present work, we focussed on the development of a recombinant DNA based methodology for the production and purification of serratiopeptidase in *E. coli* system. The prominent protein band around 50 kDa in the extracellular secretion of *S. marcescens mtcc7298* strain was the major extracellular protease of the bacteria called serratiopeptidase [15, 38]. The developed recombinant expression construct pMSrp contains the gene-specific to mature serratiopeptidase gene lacking the N-terminal pro-peptide encoding nucleotides [35] located downstream to T7 promoter and can be transcribed in *E. coli* expression strains encoding T7 RNA polymerase.

When the gene was transformed into different *E. coli* expression systems, differences in the number of transformants was evident, which could be correlated to the toxicity of the gene. *E. coli BL21 (DE3)* cells repeatedly failed during transformation, and not a single colony was formed on the plate. *E. coli C43(DE3)* cells designed by Miraux and Walker contains two mutations in − 10 region of *lac*UV5, which allows better overexpression of membrane proteins. The strain also provides stability to plasmids encoding toxic genes and allows the expression of recombinant proteins seems to be toxic in other *E. coli DE3* variants [39]. When pMSrp transformed in *C43(DE3)* cells, not only the maximum number of transformants was formed in *E. coli C43(DE3)* cells but also the transformed cells expressed serratiopeptidase protein efficiently.

Although a good number of transformants were possible in other *E. coli BL21 (DE3)* variants, viz—*E. coli BL21 (DE3)*-*pLysS, E. coli Rosetta (DE3)*-*pLysS,* they all failed at the protein expression level which could be attributed to protease associated cytotoxicity [39, 40]. The correct and maximum serratiopeptidase expression was found in *E. coli C43(DE3)* cells, which was ~ 12% of total protein expression resulting in purification of around 2–2.5 mg functionally active serratiopeptidase from 1-l of culture.

Recombinant version mature serratiopeptidase exclusively formed insoluble aggregates, i.e., inclusion bodies. Optimization of overexpression process parameters such as induction point, temperature, inducer concentration., results in enhancement of correct folding hence solubility of the protein. It also aids in the enhancement of the yield of the recombinant protein of interest [41]. Considering these two factors, viz—solubility and yield, optimization of various process parameters was carried out, which resulted in 5 times more expression of the protein providing around 20 times more yield in the form of 40–45 mg functionally active pure mature serratiopeptidase from 1-l of culture. No effect of process parameter optimization was seen on the solubility of the protein. Temperature optimization further confirmed the toxicity of the protein since no visible expression of the protein was found at lower temperatures, i.e., 25 and 18 °C. Failure in achieving the soluble protein and expression of the protein in the form of inclusion bodies could be attributed to pH, osmolarity, redox potential, cofactors, and folding differences in the intracellular microenvironment since the protein is an extracellular protein [33, 42]. Usually, when the expression of recombinant protein goes beyond 2% of the total cellular protein, it results in unregulated accumulation of the protein insoluble aggregates known as inclusion bodies [43]. Inclusion bodies formed during heterologous overexpression constitute 50–60% of the recombinant protein of interest, having the presence of fewer protein impurities [44] and could serve a better source of protein preparation if the proper method of their solubilization and refolding could be devised. A variety of proteins are successfully recovered from accumulated inclusion bodies, which are conformationally and functionally, similar to the native protein [45]. Inclusion bodies representing mature serratiopeptidase were isolated from *E. coli C43(DE3)* cells and refolded in ice-cold refolding buffer with an efficiency of around 50%. The purified mature serratiopeptidase from refolded inclusion bodies was identical to the commercially available native version on the molecular level (monomeric) as well as functional level (activity assay). Comparison of the secondary and tertiary structure of recombinant one with the commercial one through circular dichroism and intrinsic fluorescence emission suggests both are very similar. The specific activity of recombinant version is comparable to the native version, and it could serve as a recombinant biosimilar for a variety of biotechnological and industrial applications.

## Conclusions

It is nearly impossible to express proteases in *E. coli* as a functional protein due to their associated catalytic activity. Unregulated intracellular expression of proteases often results in cell death, hindrance in growth, lack of expression, degraded expression, or expression of the gene product in the form of insoluble inclusion bodies. In the present work, we specifically tried to explore the recombinant expression, purification, and physicochemical comparison of an industrially and therapeutically relevant broad specificity extracellular metalloprotease of *S. marcescens* (MTCC7298) known as serratiopeptidase. A recombinant expression plasmid pMSrp exclusive to the expression of mature serratiopeptidase lacking 16 amino acid long N-terminal propeptide was constructed by cloning the mature serratiopeptidase specific gene under T7 promoter in pET23b(+) vector plasmid. Transformation and expression in different *E. coli* expression host systems confirmed the presence of the gene is toxic for the cells causing either unsuccessful transformation (*BL21(DE3)* cells), lack of expression (*BL21(DE3)*-*pLysS* cells) or expression of the degraded product (*Rosetta(DE3)*-*pLysS* cells). Only *E. coli C43(DE3)*cells, engineered specifically for the expression of membrane proteins and toxic proteins, were expressing the protein correctly in the form of intracellular insoluble deposits, i.e., inclusion bodies.

Further optimization of various process parameters resulted in about five times more expression of serratiopeptidase than unoptimized conditions (by densitometric analysis). The overexpressed mature version of serratiopeptidase protein forms 60–65% pure inclusion bodies. Solubilization, refolding, and purification provides purified (> 98%) 45–50 mg functionally active protein from one-liter culture. The discussed recombinant approach could be a better alternative to the present traditional production strategy, considering the health hazards associated with wild strains of *S. marcescens*. The biological identity, activity, and biophysical comparison with commercially available native serratiopeptidase suggest the recombinant version could serve as a potential biosimilar for pharmaceutical and variety of industrial applications.

## Methods

### Screening of serratiopeptidase producing *Serratia marcescens* strain

*Serratia marcescens* strain (collection id-7298), collected from the Microbial Type Culture Collection (MTCC), IMTECH Chandigarh was screened for the extracellular secretion of serratiopeptidase. 0.2 μl overnight grown seed culture of *S. marcescens* was point inoculated on 1% skimmed milk agar plate following 48-h incubation at 37 °C. The identity of protease in secretion was confirmed by SDS-PAGE and further by In-gel trypsin digestion and Peptide mass fingerprinting using MALDI-TOF mass spectroscopy.

### Gene amplification and recombinant cloning

Mature serratiopeptidase gene-specific forward and reverse primers (shown in Table 3) were used for PCR based amplification of the mature serratiopeptidase (Msrp) gene, which lacks the initial 48 nucleotides encoding N-terminal propeptide. In brief-The reaction mixture contained 200 µm dNTPs (New England Biolabs, USA), 1× high fidelity buffer (Thermo Scientific, USA), and 4.5 units of Phusion polymerase (Thermo Scientific, USA) and 0.2 ng/μl genomic DNA of *S. marcescens mtcc7298* as template DNA. Amplification reaction comprised of an initial 3 min denaturation (95 °C), 35 cycles of 30 s denaturation (95 °C), 30 s annealing (T_a_ = 59 °C), and 90 s extension (72 °C) each, followed by a final 10 min extension (72 °C). A control reaction was put together containing all the components except the template DNA.

**Table 3:** Mature serratiopeptidase specific PCR primers used for gene amplification

Oligonucleotides	Sequence	Restriction site
Msrp_fwd_7298	5′-TATAATACTCATATGGCCGCGACAACC-3′	*Nde*I
Msrp_rev_7298	5′-ATGTACCTCGAGTTACACGATAAAGTCC-3′	*Xho*I

Amplified PCR product and empty pET23b(+) vector were digested for 6 h by restriction enzymes *Nde*I and *Xho*I (New England Biolabs, USA). Purified PCR product and vector after digestion were allowed to ligate at 16 °C for the overnight duration by T4 DNA ligase (New England Biolabs, USA). The ligated product was transformed into competent *E. coli DH5α* and plated on ampicillin (200 µg/ml) agar plates. Positive transformants containing the ampicillin-resistant gene (*Amp*^*r*^) were verified through colony PCR.

Restriction digestion of the recombinant plasmid was carried out to verify the size and successful insertion of *msrp* in the expression vector by visualizing the fragment, equivalent to the size of the cloned gene on 1% agarose gel. Sequencing of the expression plasmid was performed to verify the sequence using T7 promoter and terminator specific primers (Base Asia, Singapore).

### *E. coli* strain optimization

Different *E. coli BL21(DE3)* based expression host cells were screened to find an optimal expression host for pMSrp expression. Freshly prepared CaCl_2_ chemical competent cells (1.3 × 10^7^) of four different *E. coli* expression strains, viz—*E. coli BL21(DE3), E. coli C43(DE3), E. coli BL21(DE3)*-*pLysS* and *E. coli Rosetta(DE3)*-*pLysS* were transformed by 10 ng of pET23b(+) null vector and recombinant plasmid pMsrp. The positive transformants were enumerated in each strain, and the number of viable cells was taken as a criterion to select the optimal host strain. The number of transformants in *E. coli DH5*-*α* was taken as a positive control. Strains found to be successfully transformed with pMSrp were screened for optimal protein expression. Transformed cells of different *E. coli* expression strains were grown at 37 °C 220 rpm and induced by the addition of 1 mM IPTG (SRL, India) when the optical density of the culture at 600 nm reached to 0.6–0.8. Bacterial cultures were allowed to grow for 2 h at similar growth conditions before visualizing overexpression through SDS-PAGE.

### Protein expression and solubility assessment

Protein overexpression was visualized by loading normalized amount of the induced, and uninduced cell lysates on a 12% SDS_PAGE and viewed after coomassie staining. The level of overexpression was quantified through the densitometric analysis of the Gel bands. To assess the solubility of the overexpressed protein, collected cell pellets after 2 h of induction were lysed by sonication in resuspension buffer (25 mM Tris, 100 mM NaCl, and pH 7.6). The insoluble content of the lysate was separated from the soluble part by centrifugation at 10,000×*g* (10 min), 4 °C. The separated pellet from supernatant was dissolved in an equal volume of resuspension buffer supplemented with 6 M urea. Normalized amount of uninduced, induced cell lysates, soluble and pellet fraction was loaded on SDS-PAGE and analyzed after coomassie staining of the gel.

### Optimization of physicochemical parameters

For maximal expression of mature serratiopeptidase in the opted *E. coli* expression strain, different physicochemical parameters, viz—growth media, Point of induction, Inducer concentration, the temperature during overexpression, post-induction duration were optimized. The optimal value for maximal expression was determined by variating one parameter at a time. Protein expression quantification at various points of process parameters was performed through densitometric analysis of separate lanes of SDS-PAGE representing different values of the physicochemical parameter under study.

### Densitometry analysis

Coomassie blue-stained gels were imaged on Bio-Rad XR+ (USA) gel documentation unit and analyzed by the Image-lab program (Bio-Rad, USA). The relative intensity of the band was measured to quantify the overexpression of the protein. The relative percentage of the expressed recombinant protein in whole cell lysate at particular conditions was measured by selecting the whole lane and detecting the band through the ‘add band’ option (band detection sensitivity was high: 75%). A relative comparison of the overexpressed protein in different conditions was performed manually. The protein bands representing mature serratiopeptidase in each lane were selected through ‘add band’ option (band detection sensitivity was high: 75%), and the area was trimmed using ‘adjust band’ option to minimize the background. The quantity of the selected band was measured through ‘quantity tools’ and selecting one of the overexpressed protein band (lowest range) as reference. The intensity obtained was preferred as criteria to determine the optimal condition for the maximal overexpression of recombinant mature serratiopeptidase. At least three independent gels for each condition were analyzed densitometrically, and the relative mean value was plotted.

### Growth profile and specific growth rate

*Escherichia coli C43(DE3)* cells alone and transformed with pMsrp were grown at 37 °C, with and without induction under shaking condition at 220 rpm. Aliquots of 500 μl culture were withdrawn at 30 min interval until 12 h for optical density measurements. The turbidity of the samples were measured at 600 nm using Beckman UV-Spectrophotometer (USA). 1 mM IPTG was added in samples representing induced condition when the optical density of cultures at 600 nm reached 0.5–0.6. To calculate the specific growth rate constant, µ, the exponential (or logarithmic) growth phase was preferred, during this phase, the rate of increase in the number of cells was proportional to the number of bacteria present at that time. The specific growth rate constant µ was determined by fitting the data into the exponential equation using systat sigmaplot 14.0.

### Preparation of inclusion bodies, refolding, and purification

*Escherichia coli Bl21 C43(DE3)* cells were harvested after 6 h of induction at 37 °C 220 rpm by centrifugation at 10,000×*g* for 10 min at 4 °C. Separated cells were resuspended in resuspension buffer (Tris: 50 mM, NaCl-350 mM, Beta-mercaptoethanol-5 mM pH-8.0) supplemented with 500 μg/ml lysozyme and lysed through sonication (Qsonica, Cole-Parmer USA) at 25% amplitude (10 s ON 50 s OFF). The supernatant is separated from the insoluble pellet by centrifuging the solution at 15,000×*g* for 20 min at 4 °C. The separated pellet representing the mature serratiopeptidase inclusion bodies were washed twice with wash buffer (Tris: 50 mM, EDTA: 5 mM NaCl-500 mM, Glycerol: 2%, Beta-mercaptoethanol-5 mM, Triton-X-100:1.5%, Urea: 2.5 Molar pH-6.8). Remaining detergent was removed by further washing the inclusion bodies with tris buffer (50 mM, pH 7.4) and stored at − 80 °C until purification.

Inclusion bodies dissolved in 1 ml denaturing buffer (Tris: 50 mM, NaCl: 500 mM, 6 Molar Guanidinium hydrochloride, pH 7.6) were refolded by dialysis against ice-cold refolding buffer (Tris: 25 mM, NaCl: 100 mM, CaCl_2_: 5 mM, ZnCl_2_: 1 mM pH 7.6). The refolded protein fraction was separated from any misfolded/precipitated protein by centrifugation and filtration using a 0.2 µM syringe filter (MDI, India). Remaining impurities and traces of denaturant were removed through superdex G-75 Hi-Prep 10/300 GL gel filtration column (GE Life Sciences) in refolding buffer lacking the CaCl_2_ and ZnCl_2_. The purity of the fractions collected during elution was assessed through activity assay, SDS-PAGE, and Coomassie staining.

### In-gel trypsin digestion and peptide mass fingerprinting (PMF)

The protein band corresponding to the molecular weight of serratiopeptidase was manually excised, chopped into small pieces, and submerged in 25 mM NH_4_CO_3_ (Sigma Aldrich USA) containing 25 ng/μl MS grade trypsin (Pierce, Thermo Scientific USA). Digested peptides were extracted in a 1:1 mixture of 0.1% Trifluoroacetic acid (Sigma Aldrich, USA) and Acetonitrile (Sigma Aldrich, USA), mixed with matrix solution and spotted on MALDI target plate. Generated peptide mass spectra were searched in the Mascot software search engine (Matrix Science, UK) [46].

### Yield and activity measurements

The yield at different stages of refolding and purification was measured through Bradford assay, as mentioned by Kruger in a microplate format [47]. The activity of the commercial serratiopeptidase and recombinant serratiopeptidase was measured by a protease activity assay as suggested by Ruchel et al. [48] with slight modifications. Briefly, the 400 μl reaction mixture containing 1% azocasein (SRL, India) and suitably diluted protein was incubated at 37 °C for 30 min. 150 μl of 20% TCA (SRL, India) was added to stop the reaction and centrifuged for 5 min at 10,000×*g*. The supernatant was added in an equal volume of 1 N NaOH (Millipore Sigma, USA), and the absorbance was measured at 450 nm. An increase of 0.1 absorption unit after 30 min of incubation at 37 °C was taken as one enzyme unit (EU).

### Native PAGE and HPLC analysis

Native PAGE analysis was done to assess the homogeneity and purity of the purified recombinant version mature serratiopeptidase. 10% non-denaturing gel lanes were loaded with mature version serratiopeptidase along with control samples of BSA. The gel was run at constant voltage (80 V) at 4 °C and visualized after coomassie staining.

Analytical HPLC of 20 µl samples containing bovine serum albumin (BSA), lysozyme, recombinantly prepared, and commercial serratiopeptidase each containing 5 µg protein were run was carried out on Bio SEC-5 HPLC column (Agilent Technologies, USA). The elution profile for each protein was used for further comparison and analysis.

### Circular dichroism and fluorescence emission spectra

The secondary and tertiary structure profile of the purified and refolded mature version recombinant serratiopeptidase was analyzed through circular dichroism and fluorescence spectroscopy. In brief, one micromolar recombinantly prepared serratiopeptidase was scanned in Far-UV circular dichroism spectra, i.e. 200–250 nm in 1 mm path length cell using J-810 spectropolarimeter (Jasco, UK) flushed with nitrogen gas at 25 °C. Samples were scanned at a rate of 50 nm/min with a step size of 1 nm. Spectra were averaged over three scans and corrected for background by subtracting the scans of the buffer without protein.

Three independent intrinsic tryptophan fluorescence emission spectra of one micromolar protein were collected between 300 and 450 nm after excitation at 280 nm at 25 °C using carry eclipse fluorescence spectrophotometer (Agilent technologies USA) and averaged out. The circular dichroism profile and intrinsic fluorescence spectra in the same range given by one micromolar commercial version serratiopeptidase (Systopic Laboratories, India) were taken as a control for comparison.

## Supplementary information

**Additional file 1.** Additional table and figures.

## Data Availability

(1) The datasets used and/or analyzed during the current study are available from the corresponding author on reasonable request. (2) All data generated or analyzed during this study are included in this published article.

## Abbreviations

BSA: bovine serum albumin
CD: circular dichroism
*E. coli*: *Escherichia coli*
IPTG: isopropyl β-d-1-thiogalactopyranoside
pMSrp: plasmid for mature serratiopeptidase expression
*msrp*: mature serratiopeptidase gene
TFA: trifluoroacetic acid
TCA: trichloroacetic acid
RDP: *Rosetta (DE3)*-*pLysS*
