# Supplementary material for: Enhanced production of recombinant serratiopeptidase in Escherichia coli and its characterization as a potential biosimilar to native biotherapeutic counterpart
Source: Microb Cell Fact. 2019 Dec 17;18:215. doi: 10.1186/s12934-019-1267-x (PMC6918600; doi:10.1186/s12934-019-1267-x)
Supplement: Supplementary file 1 — Additional file 1. Additional table and figures. [file 12934_2019_1267_MOESM1_ESM.docx]

## Additional Material

*Table S1: Bacterial strains used in the study*

| **Bacterial Strain** | **Source** | **Relevant features** |
| --- | --- | --- |
| *S. marcescens mtcc7298* | MTCC Gene Bank, | Non-pigmented, aerobic, proteolytic strain isolated from Tasar *(Antheraea mylitta)* Gut. |
| *E.coli DH5-α* | Invitrogen | Nonpathogenic E.coli strain for cloning purposes, with high-efficiency for transformation |
| *E.coli BL21(DE3)* | Novagen, | The strain expresses T7 polymerase when induced by IPTG. It does not contain genes for lon and omp-t proteases and suitable for expression of non-toxic genes. |
| *E.coli BL21 (DE3)-pLysS* | Novagen, | Contains p15A origin pLysS plasmid, which produces T7 lysozyme to reduce basal level expression of the gene of interest provides tighter control of expression. |
| *E.coli Rosetta (DE3)-pLysS* | Novagen, | pLysS plasmid contains additional genes for the rare tRNA creating a possibility for eukaryotic protein expression. |
| *E.coli C43(DE3)* | Lucigen | Strain consists of at least one uncharacterized mutation allowing the expression of toxic and membrane proteins from all classes of organisms. |

**Figure S1: Extracellular secretion of *Serratia marcescens mtcc7298* contains 50KDa serratiopeptidase as major secretory protein. a-** Agar plates supplemented with 1% skimmed milk showing zone of hydrolysis around *Serratia marcescens* *mtcc7298* colony, an effect of extracellular secretion of protease. **b-** Representative SDS-PAGE gel lane containing medium-range protein molecular weight marker (Lane-M) and extracellular medium of Serratia marcescens mtcc7298 (10µl) after 48 hours of growth (Lane- SM) shows a significant protein band around 50kDa which is possibly the serratiopeptidase.


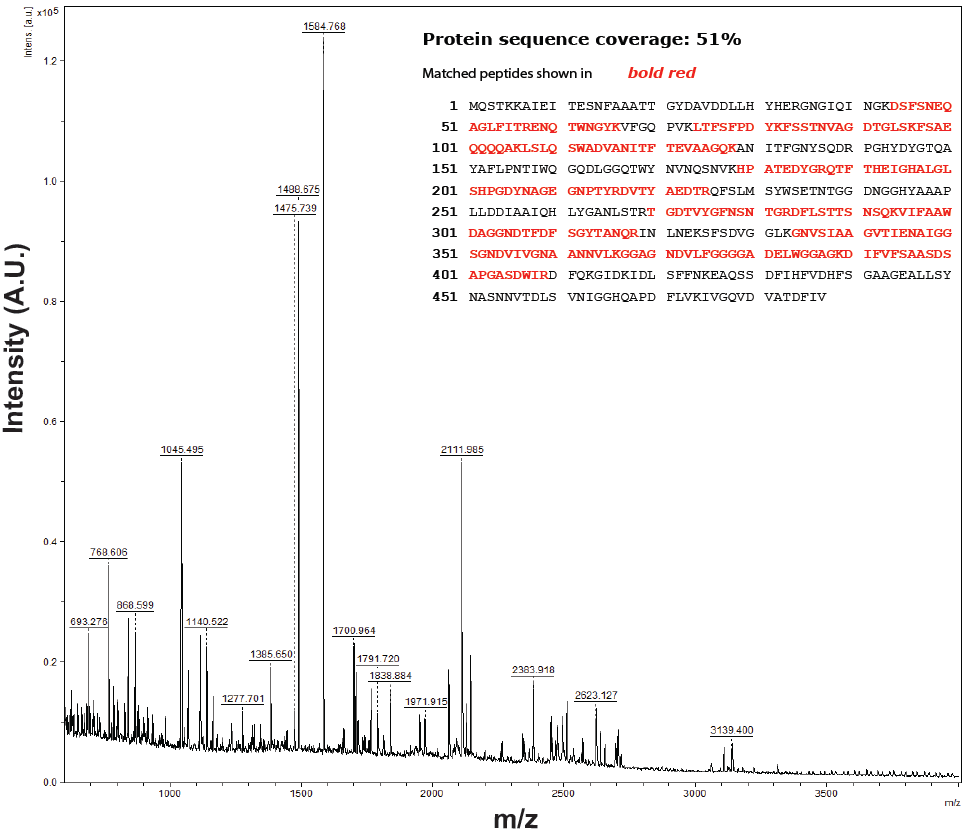


Figure S2: In gel trypsin digestion and peptide mass fingerprinting of the 50kDa protein in *Serratia marcescens mtcc7298* extracellular secretion: The secreted protein shows 51% sequence coverage with *Serratia marcescens* serratiopeptidase protein suggesting confirming the identity of the prominent band as serratiopeptidase.


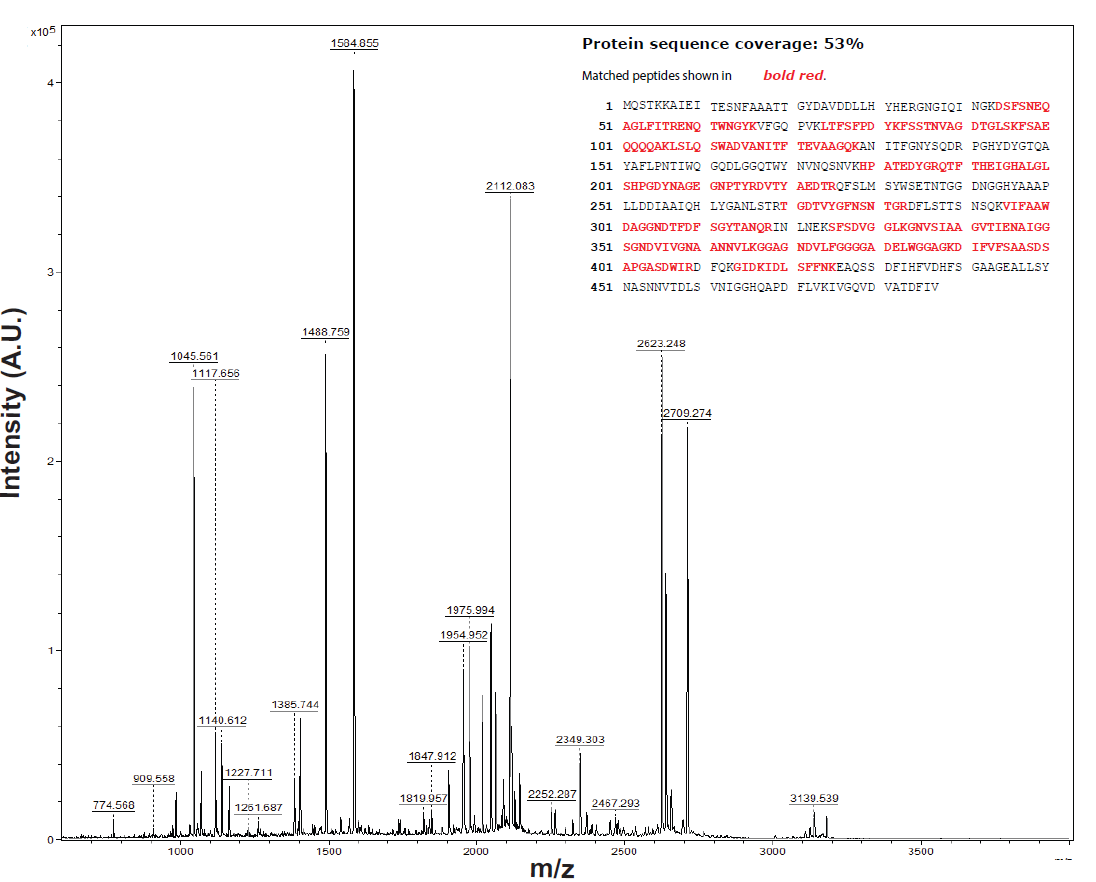


Figure S3: Peptide Mass Fingerprinting of recombinant serratiopeptidase: In-gel trypsin digestion and peptide mass fingerprinting of purified recombinant version mature serratiopeptidase shows 53% sequence coverage with serratiopeptidase protein of *Serratia marcescens. There is no matched identity with the initial 16 amino acid representing the propeptide, which confirms the cloned and purified protein is mature version of serratiopeptidase.*
